# The Institutional Library

**Published:** 1921-03-26

**Authors:** 


### THE INSTITUTIONAL LIBRARY.

**Truth about Venereal Disease.** By Mrs. Stopes, D.Sc. (Putnam's Sons. 1921. Pp. 52. Price 1s. 6d.)

There is no writer of the day upon the sexual relations of men and women who is so widely read by the educated classes as Mrs. Stopes. The present reviewer has not met a single one of her readers who understands that Mrs. Stopes is not a medical woman. This is not her fault in any way, but it results in a certain amount of misapprehension as to what she means and of the degree of authority that attaches to some of her pronouncements. She has now turned her pen to the exposition of venereal disease, and the result discloses just those qualities of earnestness, eloquence, downrightness, and good intention which her previous writings have shown that she possesses. It shows also occasional exaggerations on technical medical matters against which any competent doctor could have guarded her. It is a great pity that she does not get a medical man to read her proof-sheets, for all books of this scope intended for the lay public should be absolutely free from over-statement—heaven knows, the plain truth is startling enough! The second paragraph on page 10, for instance, contains so small a portion of the whole truth on the matter it deals with as to leave an entirely erroneous impression on the mind of the reader. The last paragraph on page 11, again, inveighs against the "audacity" of men ("silly and superficial" are adjectives used in the same connection) who blame women for refusing to have more than one child when the reason "may be" that their husbands have infected them with venereal disease. Of course, any man who did blame any woman in such circumstances would deserve all the censure Mrs. Stopes could lay her tongue to. But surely she can see that what disturbs the "publicists" she speaks so scornfully about is the deliberate and selfish refusal of motherhood by those perfectly equipped and competent for it, not the involuntary sterility of a diseased minority; and that exaggerations such as this, and that printed at the top of page 14, defeat their own object by forcing doctors to tell people that Mrs. Stopes doesn't know what she is talking about.

We have taken pains to put forward this objection in detail, because we have very warmly at heart the same object as that which Mrs. Stopes desires to achieve—namely, the safeguarding of the race against the menace of venereal diseases. It may be noted, in conclusion, that after consulting both the societies which are interesting themselves in this subject (and quarrelling violently with each other over it), Mrs. Stopes leans evidently much more nearly to the Society for Prevention of Venereal Diseases than to the Council for Combating them.

**The Diseases of the New-born.** By Dr. August Ritter von Reuss. (Bale, Sons & Danielsson. 1920. Pp. 626. Price £2 12s. 6d. net.)

This important text-book was published in Vienna, where the author held a hospital appointment, a few months before the war began. The English translation, now published, is taken, apparently, from the 1914 edition without further revision by the author, who (if all accounts of the conditions in Austria are true) must by now have widely extended his studies in the pathology of the new-born. We make the deduction implied above for the reason that foot-notes dated 1913, and explained as relating to matters arising while the proofs were being read, are retained as such—not incorporated with the general body of the text.

Dr. von Reuss draws no hard and fast line as to when a baby ceases to be "new-born"; he takes the amount of development to be expected of a normal infant in the first two weeks of life as a rough criterion, and points out that some babies may not attain to this stage in a great many weeks, where others may pass it in a few days. He makes out a good case for the intensive cultivation of this restricted corner of the field of pædiatrics, and within the limits he has chosen for himself he has undoubtedly put himself in the forefront of medical literature. His monograph is exhaustive without overloading, and scholarly without pedantry. A Teutonic love of statistics on every conceivable point is with him but a mild obsession, and fortunately does not obscure his sense of clinical reality, as it does in some German text-books. The appended bibliography is of stupendous proportions, but it is noticeable that German and Austrian authors form about 95 per cent. of those whom he has consulted. It is a little surprising to find him sticking to the original Credé method for the prevention of ophthalmia neonatorum, and not emphasising the importance of wiping away all secretion from the lids directly the head has been born. For the developed condition, too, he prefers silver nitrate to the organic silver salts, which are the favourites (and, in our opinion, rightly) in this country. Several drugs, by the way, are mentioned under proprietary names unknown here, and are consequently unidentifiable. The translation is by an unnamed writer, and has been supervised by Dr. J. D. Rolleston. It is throughout idiomatic, and indeed reads better than some medical works composed in English do; whether this is to the credit more of Dr. Rolleston or of the actual translator it is impossible to decide, but it may be guessed that the former is largely responsible. On the very first page, as it happens, there is a curious slip, whereby a case rate of 4 in 932 is described as 4.08 per cent. The illustrations are adequate without being remarkable, and there is a good index; the published price is a high one and may militate against the popularity of an excellent text-book.

**Approved Technique of the Rideal-Walker Test.** By S. Rideal, B.Sc., and J. T. Ainslie Walker (Capt. R.A.M.C.). Pp. 12. (London: H. K. Lewis & Co., Ltd.) Price 1s. net.

The title of this little brochure is self-explanatory, and for those working upon disinfectants and bactericides the reference provided is a necessary laboratory companion. With an arbitrary test, the need for strict observance of conditions laid down cannot be too strongly emphasised if comparative figures are to be of any real value. Granted that, in its unmodified form, this technique will not always provide complete indication of the clinical value of any given antiseptic, yet a test which, in the first place, gives a true measure of the simple germicidal efficiency is still of prime importance.

**Intestinal Disinfection: With a Note on Its Practical Attainment.** By J. T. Ainslie Walker, Captain, R.A.M.C. (T.F.). (London: The Universal Medical Periodicals, Ltd., 36-8 Whitefriars Street, E.C. 4. Price 6d. net.)

Considerable diversity of opinion exists as to the exact morbid effects of alimentary toxæmia, but there are sufficient well-defined clinical conditions arising therefrom to

**The Institutional Library**—(*continued*).

make a reliable non-toxic intestinal disinfectant therapeutically invaluable. The failure of such substances as salol, $\beta$-naphthol, calomel, salicylates, etc., is now generally realised, and we therefore read with great interest the author's claims for substances known respectively as trimethol (trimethylomethoxyphenol) and dimol (dimethylomethoxyphenol). The Rideal-Walker co-efficient of both lies at 35 to 40, but, so far, clinical experience gives preference to the former. The author's small brochure is very easy reading, and his experience already gained with these products suggests that they might well be given an extended trial in institutional practice.

**The Surgical Aspects of Dysentery.** By Zachary Cope, B.A., M.D., M.S., F.R.C.S. (The Oxford Medical Publications, Henry Frowde, and Hodder & Stoughton.) Price 12s. 6d. net.

This work embodies portions of the Hunterian Lecture which originally appeared in the *Lancet*, and the author, as surgical specialist with the Mesopotamian Expeditionary Force, 1916-19, is able to speak from a great fund of experience. Every big campaign produces its epidemic of dysentery, and the late war proved no exception to the rule. Surgical complications may not, in future, be common in dysentery, with improved therapeutic measures now available. But the large numbers of latent subacute and chronic post-war cases, now inevitably distributed in temperate as well as tropical areas, make efficient surgical knowledge of the condition highly necessary. At present the literature of the subject is both scanty and scattered, and Mr. Cope, by clearly recording his successes, difficulties—and mistakes—with unbiased candour, has supplied an obvious need. Both he and the publishers are to be congratulated upon the result of their respective efforts.

**Text-book of Tracheo-Bronchoscopy.** By Dr. M. Mann. Translated by A. R. Moodie, M.D. (Bale, Sons, and Danielsson. 1920. Pp. 291. Price 31s. 6d. net.)

This must be, we take it, the first translation of an important German textbook since the war, and if there are any fastidious patriots who feel inclined to sniff because the author is a German, at least let them remember *fas est et ab hoste doceri*. Frankly, there is no living Englishman who could, to the best of our belief, have written this book: an unpleasant thought, perhaps, but not one to be blinked. Dr. Mann's purpose goes much further than to produce an account of how to use tracheo-bronchial illuminated tubes so as to remove foreign bodies from the air-passages, though to this subject he pays, of course, great attention. He pleads for tracheo-bronchoscopy that it is capable of an important rôle in the diagnosis (and occasionally in the treatment) of pulmonary and other intra-thoracic diseases. If this premise be granted—and he produces very strong evidence to prove it—it is difficult to refuse assent to his deduction that the general surgeon and the general physician ought to acquire the rather complicated though not really difficult technique of tracheo-bronchoscopy instead of leaving it to a handful of laryngologists. Dr. Mann, in other words, can dive below externals down to first principles. It may be some years possibly before the profession wholly adopts his view about this, but it is to be hoped that serious consideration will be given to it in this country. Such a consummation Dr. Moodie and the publishers have done their best to promote: their parts in the task of presenting the most up-to-date developments of tracheo-bronchoscopy have been very ably played, and they deserve a special word of thanks accordingly. Bibliophiles may be interested to note that although the page of the book is as large as that of an average quarto (indeed, larger than most of the early quartos), yet the book is technically what is called 16mo: that is, the "signature" occurs only every thirty-two pages, showing that sixteen pages are printed at the same moment by each press.

**Woman.** By Magdeleine Marx. (Allen. 1921. Price 7s. 6d. net.)

It is nearly always a mistake to pitch expectation too high; even the Wonders of the World will barely stand the strain of over-anticipation. This mistake in tactics is committed by the publishers when they enclose Miss (or Mrs.) Marx's masterpiece in a wrapper covered with flamboyant laudations by various French (and two English) authors. One expects an answer to the riddle of the Sphinx: something at once profound, catholic, kindly, tender, true. And all we get is a series of *anacolutha*, of half-finished sentences ending in rows of dots, of half-intelligible epigrams separated by hiatuses. Perhaps that is the secret the author is letting us into—that woman is a creature only to be expressed in lost chords and broken melodies, an irresponsible, indefinite, inconsequent medley of half-formed ideas and half-expressed sentimentalities. If this is what Magdeleine Marx means, she apparently also means that polyandry is normal and that maternal affection is not, or is at least less so than the former; for the heroine, after losing both her husband and her paramour in the war, is left at the end of the book expressing the confident hope that presently she will find another mate—whether with benefit of clergy or without she does not explain. Heaven forbid that any man should presume to think he understands the female of the species; that she is more deadly than the male we have poetic authority for believing, and Magdeleine Marx's presentment of her suggests that this is an under-statement of the facts.

**A Manual of Public Health.** (10s. 6d. net.)

**A Manual of Medical Jurisprudence and Toxicology.** (12s. 6d. net.) Both by W. G. Aitchison Robertson, M.D., D.Sc., F.R.C.P.E., F.R.S.E. Fourth edition. (London: A. & C. Black, Ltd., Soho Square, W. 1.)

For the new edition of this well-known work it has been decided to separate the text into the above two volumes. Apart from obviously increased care of sectional reference, the two spheres dealt with have so little in common that students will undoubtedly approve of the change, more especially as separate lecturers frequently deal with these branches, and as they are generally studied at separate times. We have previously expressed approval of the masterly way in which the author has carried out a seemingly impossible condensation of all the information necessary for the average public-health examinations. For this latest edition we can readily repeat our praise.

**This Concerns You.** By S. G. Moore, M.D., Ch.B., D.P.H. (Published by the St. Catherine Press, Stamford Street, Waterloo, London, S.E. 1. Price 2d. net.)

This little book forms a useful addition to popular literature urging a worldwide campaign against impure milk. It has been written for the American philanthropist, the Hon. Nathan Straus, whose distributions of Pasteurised milk in New York and great efforts to combat impure milk dangers in the U.S.A. have earned well-deserved commendation. The least that can be said for his present pamphlet is that it contains some remarkably sound advice.

